# Computer-aided therapeutic diagnosis for anorexia

**DOI:** 10.1186/s12938-020-00798-9

**Published:** 2020-06-19

**Authors:** Dominik Spinczyk, Mateusz Bas, Mariusz Dzieciątko, Michał Maćkowski, Katarzyna Rojewska, Stella Maćkowska

**Affiliations:** 1 Faculty of Biomedical Engineering, Silesian University of Technology, 40 Roosevelta, 41-800 Zabrze, Poland; 2 SAS Institute Sp. z o.o, Gdańska 27/31, 01-633 Warsaw, Poland; 3 Faculty of Automatic Control, Electronic and Computer Science, Silesian University of Technology, 16 Akademicka, 44-100 Gliwice, Poland; 4 Faculty of Pedagogy and Psychology, University of Silesia in Katowice, 53 Grażyńskiego, 40-126 Katowice, Poland

**Keywords:** Affective verbal stimuli, Anorexia nervosa, Emotion, Nencki Affective Word List, Sentiment analysis, Sentiment dictionary, Text classifiers, Text mining

## Abstract

**Background:**

Anorexia nervosa is a clinical disorder syndrome of the wide spectrum without a fully recognized etiology. The necessary issue in the clinical diagnostic process is to detect the causes of this disease (e.g., my body image, food, family, peers), which the therapist gradually comes to by verifying assumptions using proper methods and tools for diagnostic process. When a person is diagnosed with anorexia, a clinician (a doctor, a therapist or a psychologist) proposes a therapeutic diagnosis and considers the kind of treatment that should be applied. This process is also continued during therapeutic diagnosis. In both cases, it is recommended to apply computer-aided tools designed for testing and confirming the assumptions made by a psychologist. The paper aims to present the computer-aided therapeutic diagnosis method for anorexia. The proposed method consists of 4 stages: free statements of a patient about his/her body image, the general sentiment analysis of statement based on Recurrent Neural Network, assessment of the intensity of five basic emotions: happiness, anger, sadness, fear and disgust (using the Nencki Affective Word List and conversion of words to their basic form), and the assessment of particular areas of difficulties—the sentiment analysis based on the dictionary approach was applied.

**Results:**

The sentiment analysis of a document achieved 72% and 51% of effectiveness, respectively, for RNN and dictionary-based methods. The intensity of sadness (emotion) occurring within the dictionary method is differentiated between control and research group at the level of 10%.

**Conclusion:**

The quick access to the sentiment analysis of a statement on the image of patient’s body, emotions experienced by the patient and particular areas of difficulties of people prone to the anorexia nervosa disorders, may help to establish the diagnosis in a very short time and start an immediate therapy. The proposed automatic method helps to avoid patient’s aversions towards the therapy, which may include avoiding patient–therapist communication, talking about less essential topics, coming late for the sessions. These circumstances can guarantee promising prognosis for recovering.

## Background

Anorexia nervosa is a clinical disorder syndrome of the wide spectrum without a fully recognized etiology. Anorexia is a continuation of disorders, which were the result of the synergistic effect of biological, psychological, and sociocultural factors [1–4]. When we consider disorder spread, chronic nature, high mortality and the influence of the disorders on the patients’ activity and their families, anorexia seems to be the most dangerous disease among girls and young women in highly developed countries [5, 6].

Presently, children, women aged 30, or even women experiencing menopause, are increasingly diagnosed with anorexia [7]. However, the age group that is notably predisposed to suffer from eating disorders is adolescents. It may result from a physiological increase in body weight, which is essential to start puberty and simultaneously increased interest in the body [8]. It makes that girls intensively start to think of themselves as unattractive, and then take some actions aimed at changing their bodies to look more attractive.

People suffering from eating disorders give too much meaning to their body (its weight and shape) that is manifested by an excessive interest in appearance and food. As a result, it becomes the most crucial core value [8]. Several various factors influence shaping this specific attitude. The most essential are the patients’ experiences and personality traits. According to numerous research, patients have a distorted image of their body, think they are fat even when their weight is correct [9]. In consequence, they impose themselves the food restrictions. Their self-esteem is based on achieving and maintaining the underweight. Specific food preferences among girls with anorexia—and other behaviors responsible for reducing the weight—majorly contribute to emaciation. Hence, it can be deduced that they constitute a specific way of interfering with the body so that it could be as close as possible to the ideal image accepted by the patient.

The body of people suffering from anorexia is also the source of very extreme emotions, which secondarily maintain its distorted image. The distorted body image and the fear against getting on the weight are referred to as the basic symptoms of anorexia [10]. They are the principal elements of the clinical picture of this disease. The early diagnosis of the intensified negative emotions towards the body and their identification (fear, disgust, auto-aggression, shame, etc.), can help to take appropriate treatment.

The fundamental issue in the clinical diagnostic process is to detect the causes of the disease (e.g., my body image, food, family, peers), which the therapist gradually comes to by verifying assumptions using proper methods and tools in the diagnostic process. When anorexia is diagnosed, a clinician (a doctor, a therapist or a psychologist) proposes a therapeutic diagnosis and considers the kind of treatment. This process is also continued during therapeutic diagnosis. In both cases, a very beneficial is applying computer-aided tools designed for testing and confirming the assumptions made by a psychologist.

In the professional literature, one can find studies that indicate the relationship between the patient’s psychiatric condition (suffering, for example from anxiety, depression, neuroses) and his/her thoughts, expressions [11–13]. Most of these studies use methods for Natural Language Processing—NLP to diagnose the potential areas of psychiatric disorders. NLP, as a subfield between linguistics and artificial intelligence, supports the clinical diagnosis and helps to shorten the time needed for disease identification.

The studies concerning the automatic identification of people with eating disorders, where the focus was on clinical diagnosis, can also be found in the literature. For instance, in a study [14], NLP methods were used for diagnosing binge-eating disorders (BED). The identification was possible thanks to the medical notes prepared by a doctor during therapeutic sessions with a patient. Here, the conversation between a patient and a therapist was supervised by a doctor. In fact, it eased the disorder identification.

The methods for automated diagnosis of anorexia presented in the references, also apply the machine learning approaches. In [15], authors present the results of using classifiers: Adaboost, logistic regression, Support Vector Machine, and Random Forest to identify and classify the comments posted on the internet service Reddit as the comments of people suffering from anorexia and the healthy ones. The classifiers’ learning process was made on 80% of the comments of both healthy and sick people, whereas the validation process was made on the remaining 20% comments. Additionally, in the case of using various text features within the Natural Language Processing, also the quality of classification was checked. The characteristic features obtained via Bag-of-Words and Unified Medical Language System [16] were used to achieve this goal. For classification assessment, the measures of sensitivity, precision, and *F* measure were used. In the case of testing the Bag-of-Words features, the values of the measures of classifiers assessment were above 90%. At the results evaluation phase on the data set not constituting the learning set, for the same classifiers and Bag-of-Words features, the values for precision were 80–86%. However, the sensitivity measure took the values only around 15% for Random Forest classifiers and up to 56% for Support Vector Machine.

In terms of machine learning for anorexia diagnosis, also Convolutional Neural Network (CNN) is used. Paper [17] discusses the use of a classifier based on CNN and weighting words in a document with Inverse Document Frequency. The methods, as mentioned earlier, are applied for determining, based on the comments left on Twitter or Reddit, whether a person is in the risk group of anorexia. For the evaluation process: precision, sensitivity, F measures, and the error for early detection of the risk were employed [18]. The classification assessment results are as follows: the precision value was at the level of 60%, sensitivity 76%, F measure—76%. In case of an error of early risk detection, the values were between 11.14% and 13.65%, which means that at least 11.14% of a person’s notes must be passed to the network input to classify the risk of anorexia correctly. The authors, in the method proposed below, decided to use a deep recursive network to classify the sentiment of the patient’s body image. It was since they provide the best quality results compared to other classifiers.

According to our knowledge, there are no methods of processing the natural language for automatic language analysis to support a psychologist’s work. Most of the research found in the literature focuses mainly on the patient’s statements. The proposed research, on the other hand, intends to determine the quantitative analysis of the patient’s health condition based on his/her notes (statements), which is a complete novelty.

The current paper focuses on automatic computer-aided therapeutic diagnostics for anorexia, dedicated to patients with the confirmed clinical diagnosis. The paper assumes that a patient describes his/her body image. This task can be made at the patient’s home to improve the patient’s comfort. The presented methodology supports the therapist by automatic sentiment analysis of a patient towards his/her body by identifying the intensity of five basic emotions. It includes happiness, anger, sadness, fear and disgust, and six particular areas of difficulties: self-esteem, acceptance of the assessment of the environment, experienced emotions, autoimmune, the functioning of the body, body image. The results of sentiment analysis can help the psychologist to verify the hypothesis made during the therapeutic procedures.

The proposed automatic method helps to avoid patients’ repulsions towards the therapy. In many cases, the patient tends to avoid communication with the therapist, talks about less essential topics, or even comes late for the sessions [19, 20]. American Psychiatric Association recommends three-phase treatment of anorexia: nutritional rehabilitation phase, psychosocial interventions, medications, and other somatic therapies [21]. The proposed method refers to the first phase of treatment, which requires extensive non-invasive therapeutic diagnosis.

The paper was designed in the following way. Methods section describes in detail the following steps of the proposed approach: the criteria of including/excluding the patient in the test, method of sentiment analysis based on RNN, the analysis of emotion intensity using the Nencki Affective Word. It also refers to the previously proposed analysis of particular areas of difficulties. The Results section presents the evaluation of sentiment analysis results obtained by two methods. The first one is the previously developed dictionary method, and the other is a new method that uses Recurrent Neural Network (RNN). It was proved that the results were improved thanks to applying the new method. The further part shows the measures of emotion intensity possible to achieve and the way how the presented method can support therapeutic diagnosis of a particular patient. It includes an anonymous notation made by a patient on his/her body image, the results of sentiment analysis, emotions, specific difficulties, and the analysis prepared by a psychologist.

## Results

**Fig. 1:**
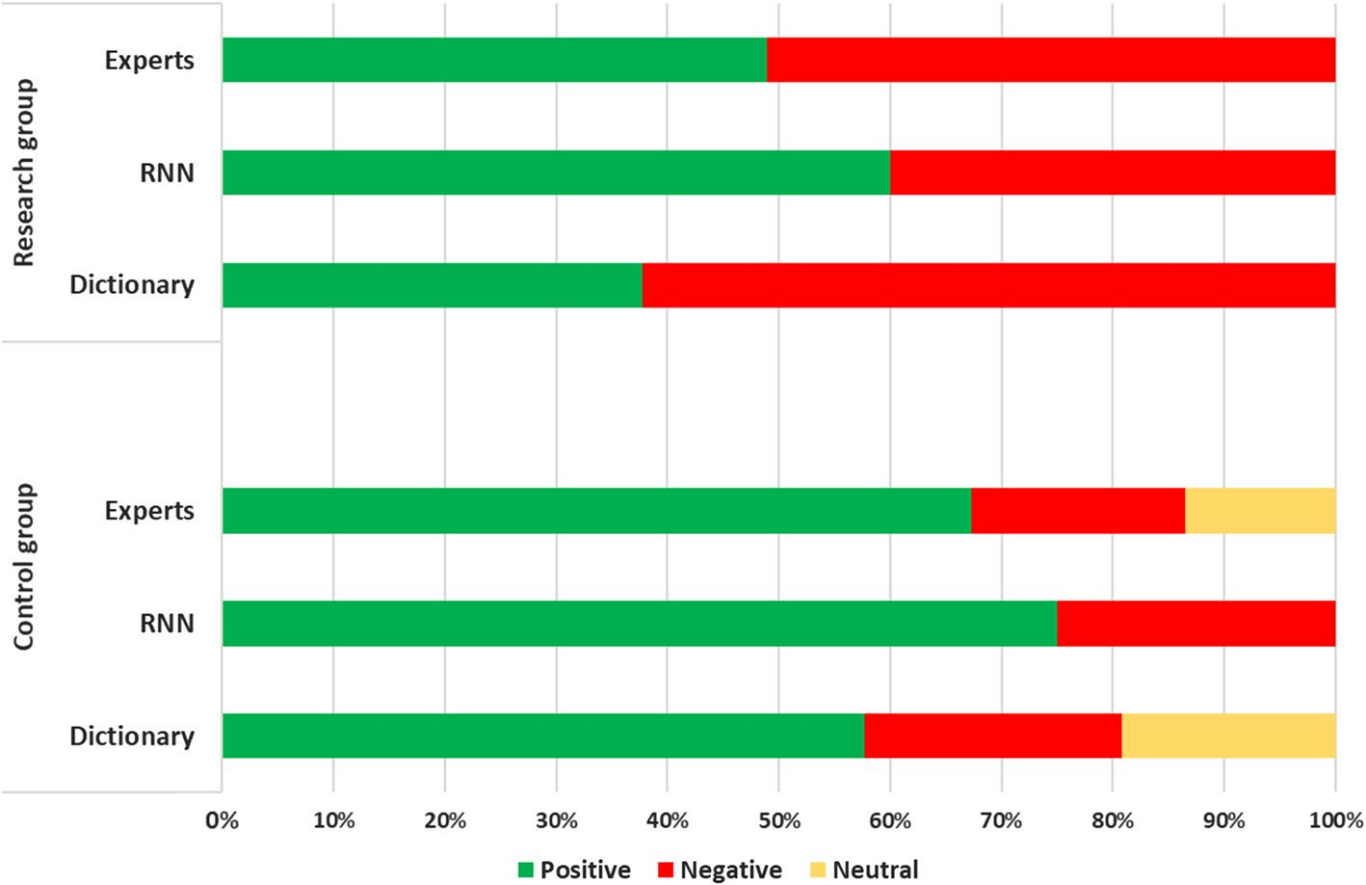
The comparison of sentiment analysis in the research and control group

**Fig. 2:**
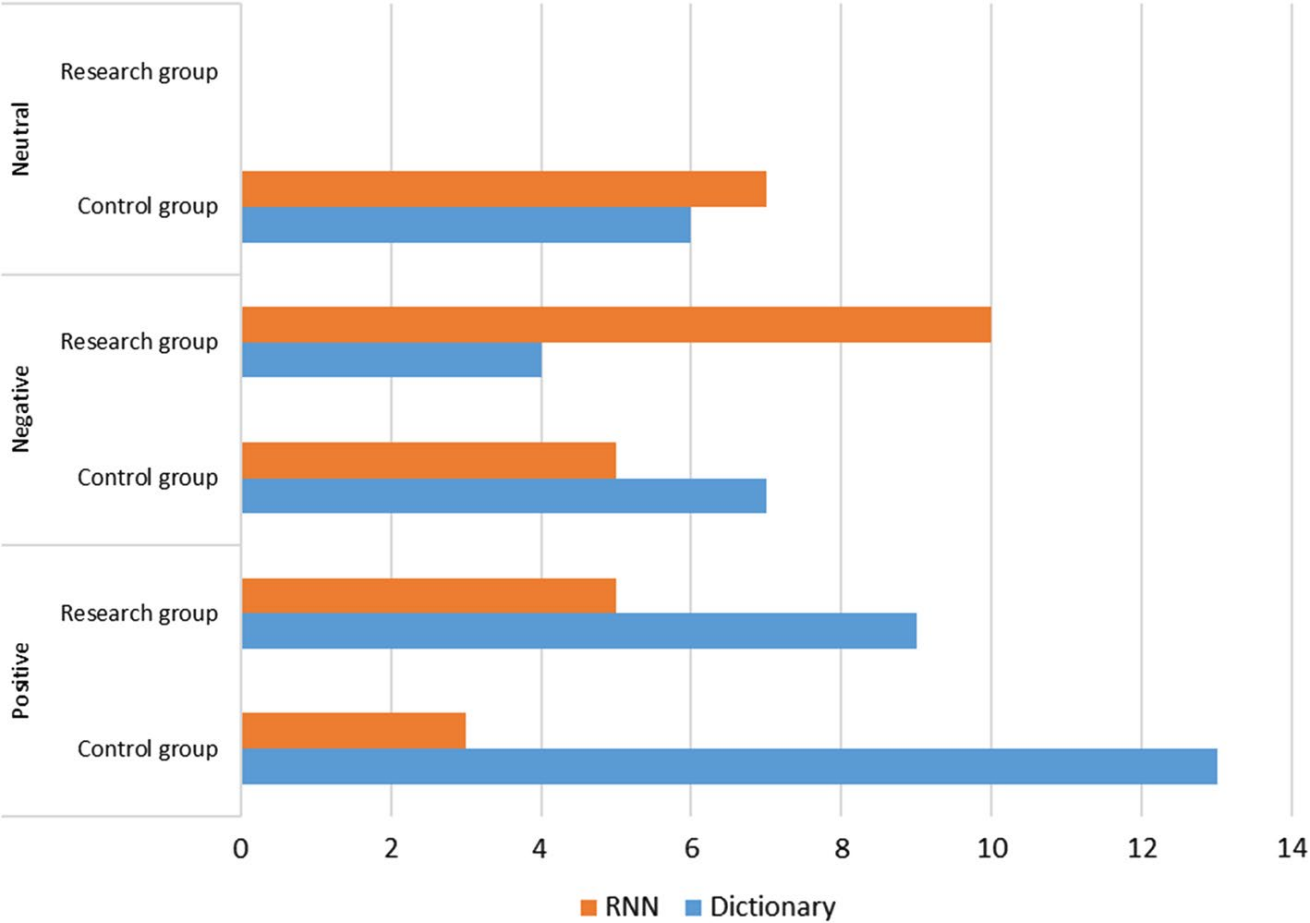
The chart presenting the sentiment classification errors in research and control group

The results of experiment E1 in the research group and the control group are presented in Table 1. A summary of the results is shown in Fig. 1. The comparison of sentiment analysis in the research and control group is presented in Fig. 2 and Tables 2, 3, 4.

**Table 1:** The sentiment analysis of a statement referring to “The image of the body”

	Document ID	Class label (expert)	Class label (RNN method)	Class label (dictionary method)	Probability of belonging to the indicated class in the RNN method	Quantitative value of sentiment in the dictionary method
Pathological subjects (research group)	1	1	**1**	**1**	0.58	0.33
	2	1	*− 1*	*− 1*	0.93	− 0.82
	3	1	*− 1*	*− 1*	0.81	− 0.43
	4	− 1	*1*	**− 1**	0.65	− 0.60
	5	− 1	**− 1**	**− 1**	0.88	− 0.67
	6	− 1	**− 1**	**− 1**	0.63	− 1.00
	7	1	**1**	**1**	0.72	0.33
	8	− 1	**− 1**	**− 1**	0.68	− 1.00
	9	1	**1**	**1**	0.58	0.33
	10	1	**1**	**1**	0.68	1.00
	11	− 1	*1*	**− 1**	0.79	− 0.60
	12	− 1	**− 1**	**− 1**	0.64	− 1.00
	13	− 1	*1*	**− 1**	0.60	− 1.00
	14	− 1	*1*	**− 1**	0.51	− 0.71
	15	1	**1**	**1**	0.81	0.29
	16	1	**1**	**1**	0.82	0.33
	17	1	*− 1*	*− 1*	0.96	− 0.45
	18	− 1	**− 1**	**− 1**	0.99	− 0.33
	19	− 1	*1*	**− 1**	0.98	− 0.20
	20	− 1	*1*	**− 1**	1.00	− 1.00
	21	1	**1**	**1**	0.92	1.00
	22	1	**1**	*− 1*	0.57	− 1.00
	23	1	**1**	*− 1*	0.65	− 0.67
	24	− 1	**− 1**	**− 1**	0.67	− 1.00
	25	− 1	**− 1**	*1*	0.95	1.00
	26	1	**1**	**1**	0.75	1.00
	27	− 1	*1*	**− 1**	1.00	− 0.33
	28	− 1	**− 1**	**− 1**	0.87	− 0.50
	29	− 1	**− 1**	*1*	0.91	1.00
	30	1	**1**	*− 1*	0.68	− 1.00
	31	1	**1**	**1**	0.82	0.60
	32	1	*− 1*	*− 1*	1.00	− 0.45
	33	− 1	**− 1**	**− 1**	0.91	− 0.30
	34	− 1	*1*	**− 1**	0.72	− 0.33
	35	− 1	*1*	**− 1**	0.98	− 1.00
	36	1	**1**	**1**	0.84	0.33
	37	− 1	**− 1**	**− 1**	0.99	− 1.00
	38	1	**1**	**1**	0.95	0.33
	39	− 1	**− 1**	**− 1**	0.72	− 1.00
	40	− 1	**− 1**	*1*	0.85	0.33
	41	1	**1**	**1**	0.95	1.00
	42	− 1	*1*	**− 1**	0.99	− 0.33
	43	− 1	**− 1**	**− 1**	0.50	− 0.60
	44	− 1	*1*	*1*	0.96	1.00
Healthy subjects (control group)	1	0	*− 1*	*1*	0.82	0.33
	2	1	**1**	**1**	0.96	1.00
	3	1	**1**	**1**	0.99	1.00
	4	− 1	*1*	**− 1**	0.98	− 0.14
	5	1	**1**	*− 1*	1.00	− 1.00
	6	0	*1*	*0*	0.92	0.00
	7	1	*− 1*	*− 1*	0.57	− 0.08
	8	0	*− 1*	*− 1*	0.65	− 0.33
	9	0	*1*	*1*	0.67	1.00
	10	1	**1**	**1**	0.95	1.00
	11	0	*− 1*	*− 1*	0.75	− 1.00
	12	1	**1**	**1**	1.00	0.20
	13	− 1	*− 1*	*1*	0.87	0.50
	14	1	**1**	*− 1*	0.91	− 0.33
	15	1	**1**	**1**	0.68	1.00
	16	− 1	**− 1**	*0*	0.82	0.00
	17	0	*1*	**0**	1.00	0.00
	18	1	**1**	**1**	0.91	0.33
	19	1	**1**	**1**	0.72	0.67
	20	1	**1**	*− 1*	0.98	− 1.00
	21	1	**1**	**1**	0.84	1.00
	22	1	**1**	**1**	0.99	0.20
	23	1	**1**	*− 1*	0.95	− 0.53
	24	− 1	*1*	*1*	0.72	0.60
	25	1	**1**	**1**	0.85	0.33
	26	1	**1**	*0*	0.95	0.00
	27	1	**1**	**1**	0.99	0.50
	28	1	**1**	**1**	0.50	0.33
	29	1	**1**	*0*	0.96	0.00
	30	1	**1**	*0*	0.99	0.00
	31	1	**1**	*0*	0.51	0.00
	32	− 1	*1*	*0*	0.94	0.00
	33	1	**1**	**1**	1.00	0.20
	34	1	**1**	*− 1*	0.79	− 0.33
	35	1	**1**	**1**	0.94	1.00
	36	1	*− 1*	**1**	0.80	1.00
	37	− 1	**− 1**	**− 1**	0.67	− 1.00
	38	− 1	*1*	*1*	0.68	1.00
	39	− 1	**− 1**	*1*	0.71	0.33
	40	1	**1**	**1**	0.95	0.33
	41	1	**1**	**1**	0.99	0.33
	42	1	**1**	*− 1*	0.97	− 0.14
	43	1	**1**	*0*	1.00	0.00
	44	1	**1**	*0*	0.57	0.00
	45	0	*− 1*	*− 1*	0.50	− 0.14
	46	1	*− 1*	**1**	0.94	1.00
	47	1	**1**	**1**	0.91	0.50
	48	1	**1**	**1**	0.74	0.50
	49	− 1	**− 1**	*1*	0.65	1.00
	50	1	**1**	**1**	1.00	0.33
	51	− 1	**− 1**	*1*	− 0.90	0.50
	52	1	**1**	**1**	1.00	0.60

Bold font indicates that the prediction matches the expert’s opinion; italics indicate that the prediction does not match the expert’s opinion

**Table 2:** Comparison of the number of sentiment classification errors [%]

Kind of group	Sentiment	Methods	
		RNN	Dictionary
Research group	Positive	11.63	20.93
	Negative	23.26	9.30
	Neutral	0.00	0.00
	Percentage of mistake	34.88	30.23
Control group	Positive	5.77	25.00
	Negative	7.69	15.38
	Neutral	13.46	9.62
	Percentage of mistake	26.92	40.38
Percentage of classification errors		30.53	35.79

**Table 3:** True-positive rate, true-negative rate, positive predictive value, negative predictive value and F-score sentiment classification quality measures of RNN classifier and dictionary method for positive, neutral and negative sentiment labels and overall means of presented measures

RNN						Dictionary				
	TPR	TNR	PPV	NPV	F1-score	TPR	TNR	PPV	NPV	F1-score
Positive label	0.87	0.57	0.73	0.77	0.79	0.64	0.71	0.74	0.60	0.69
Neutral label	–	1.00	–	0.93	–	–	0.91	–	0.94	–
Negative label	0.57	0.82	0.65	0.77	0.61	0.66	0.73	0.58	0.79	0.61
Mean	0.72	0.80	0.69	0.82	0.70	0.65	0.78	0.66	0.78	0.65

**Table 4:** Statistical significance results of Wilcoxon matched-pairs signed-ranks test

	Diseased (research group) and healthy (control group) combined		
	Expert labels	Dictionary labels	RNN labels
Expert labels	**X**		
Dictionary labels	***0271***	**X**	
RNN labels	***0176***	*0034*	**X**

Bolditalics indicate a statistically significant agreement between the predictions received and the expert’s opinion

As the given data show, it seems to be obvious that the sentiment analysis using the RNN is more effective than the dictionary method (the classification error is an almost twofold increase). The differences result from the way of analyzing the document. RNN takes into account the entire document content, including the word order. The dictionary method, on the other hand, focuses only on the number of words—not their order.

The results of experiment E2 (analysis of emotions) in the research and control group are shown in Table 5.

**Table 5:** Analysis of five basic emotions: happiness, anger, sadness, fear and disgust referring to the statement “The image of one’s body”

	Document ID	Happiness	Sadness	Fear	Disgust	Anger
Pathological subjects (research group)	1	1.00				
	2		0.82	0.23		
	3	0.75		0.076		
	4					
	5	0.84		0.095	0.12	
	6	0.53				
	7	0.75		0.095		
	8	0.59				
	9	1.00				0.33
	10	0.67				
	11					
	12		0.53			
	13					
	14					
	15	0.55	0.53			
	16	0.78				
	17		0.82	0.24		
	18	0.75		0.076		
	19					
	20					
	21	0.84		0.089		
	22	0.53				
	23	0.75		0.095		
	24	0.59				
	25	1.00				0.32
	26	0.67		0.095		
	27					
	28		0.53			
	29					
	30					
	31	0.78				
	32		0.82	0.24		
	33	0.75		0.076		
	34					
	35				0.12	
	36	0.84		0.089		
	37	0.53				
	38	0.75		0.095		
	39	0.59				
	40					0.32
	41	0.67				
	42					
	43		0.54			
	44					
Healthy subjects (control group)	1	0.53				
	2	0.64				
	3					
	4					
	5	0.60				
	6	0.53				
	7					
	8					
	9					
	10		0.54			
	11	0.87				
	12	0.81				
	13					
	14					
	15					
	16	0.89	0.54		0.31	0.32
	17	0.67				
	18	0.53				
	19	0.81				
	20	0.67				
	21			0.32		
	22					
	23					
	24					
	25					0.32
	26					
	27	0.89				
	28					
	29					
	30	0.72		0.03		
	31					
	32	0.91				
	33					
	34			0.08		
	35	0.73				
	36	0.98				
	37					
	38	0.90		0.10		
	39					
	40					1.00
	41	0.91				
	42	0.71				
	43	0.75				
	44	0.60				
	45					
	46					
	47					
	48	0.78				0.21
	49					
	50	0.69				
	51					
	52	0.53				

The results of the analysis, referring to emotions, clearly indicate that it is impossible to determine the level of intensity of a given emotion for an individual patient. It is due to the fact that sentences are short and do not contain words occurring in the dictionaries. Based on the collected results, it can be concluded that the difference in the intensity of sadness between research and control group is about 10%.

### The exemplary analysis of an individual patient

The developed method applied for an individual patient, and the patient’s medical review is presented below. Example statement—a note prepared by the patient:

*“How did it start? Very simply, One day I understood that since nobody accepts me, I will accept myself, I started losing weight, I was never overweight* - *I was just a chubby, the problem is that I hate being fat! At the beginning of losing weight, I ate very little, After a short time, I realized that if I do not eat, I feel incredible satisfaction* - *I feel so strong and persistent. So in the beginning I gave up sugars, butter, and fats, Now my day meal is a salad. I know I need help, I don’t feel good anymore from not eating* - *I feel caught in a cage without retreat”*.

**Fig. 3:**
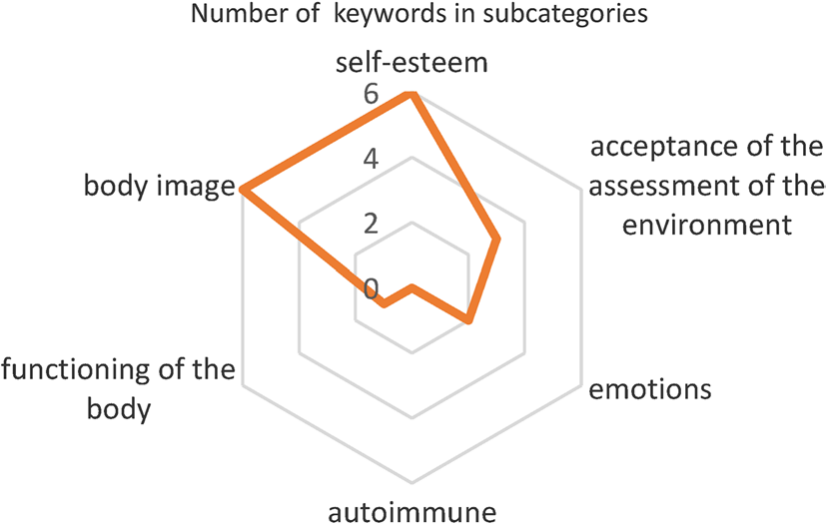
The chart of detailed areas of difficulties for an individual patient from the research group

Both methods of sentiment analysis showed an extremely negative sentiment at the patient (RNN method: class: − 1 with probability: 0.68; dictionary method: class − 1, the quantitative value of sentiment: − 1). The estimated level of sadness intensity: 0.8 and fear: 0.24. The chart, including the specific areas of difficulties for an individual, is presented in Fig. 3.

Psychologist’s analysis: The quoted words of a patient with AN includes statements suggesting the occurrence of typical, axial symptoms of eating disorders such as fear against getting fat, avoiding products with animal fat, satisfaction from maintaining the control and a restricted diet, losing the weight, feeling of helplessness when the symptoms increase. The obtained results using the proposed method coincide with the psychologist’s opinion.

## Discussion

The developed method helped to establish a quick therapeutic diagnosis in 40 out of the 44 cases. The intensity of negative sentiment constitutes a set of hints for a psychologist. Recognized emotional intensity helps to lead the conversation so that to find the reasons for experiencing such strong emotions. Indicating the areas of difficulties was particularly useful, which may reduce the necessity of searching by psychologist issues that are the most problematic for a patient. In extreme cases, the therapist tries to avoid topics related to the areas of difficulties. In less severe cases, on the other hand, it is suggested to start the therapy from the detected areas of difficulties. In the 3 remaining cases, because of the severity of the disease, it was decided to apply pharmacological treatment in the first phase of the therapy.

The occurrence of other computer-aided methods for anorexia diagnosis in the literature is quite poor. The proposed method seems to be an interesting alternative for verifying diagnostic hypotheses, mainly thanks to the quick access to behaviors and emotions related to body image and nutrition. It can be achieved by triggering various connotations via the introductory sentence.

This method does not include phrases or sentences often used in the medical interview, which may intensify defense mechanisms connected with neglecting the diagnosis. Therefore, the patient can feel more comfortable and safe. The flexibility in the statement length, type of phrases, words, and form used by the patient also helps to express easily the emotions being experienced, inner conflicts, and the patient’s attitude to her own body.

The developed automatic method contributes to reducing patient’s dislike towards the therapy, which can be depicted as avoiding patient–therapist communication, leading the conversation to the less essential topics or even coming late for the sessions [19, 20]. American Psychiatric Association established three-phase treatment standards for anorexia: nutritional rehabilitation, psychosocial interventions, medications, and other somatic treatments [21]. The proposed method focuses on the first phase of treatment, where the extensive non-invasive diagnostics is required. This method can be applied several times in the treatment process providing essential comparative data.

Despite all the advantages of the proposed method obviously, it has some weak points. Some inconvenience for dictionary methods is language mistakes occurring in texts. However, a method for automatic error correction based on Damerau–Levenshtein length was proposed. It was proved that this method could correct above 95% of writing errors occurring in Polish texts [49].

In case of very short statements (single words), the sentiment can be misestimated, whereas for longer statements, its effectiveness if satisfying. A similar situation refers to the analysis of the emotions and detailed areas of difficulties. Sometimes the therapist has problems with analyzing long statement made by a patient. Then, computer-aided support seems to be pretty helpful. Another useful step is to make some comparisons of a particular patient’s notes during the therapy, which could determine the further direction for the method assessment. The sentiment analysis of anorexia words is overstretched by the subjective meaning of particular terms [50, 51]. Therefore, essential work is to aggregate the results of various methods of sentiment analysis. It may indicate further research direction.

## Conclusions

The article presents the concept of computer-aided therapeutic diagnosis of anorexia. It includes an assessment of body image sentiment, analysis of the intensity of five basic emotions: happiness, anger, sadness, fear, and disgust. Moreover, it indicates potential six areas of difficulty: self-esteem, acceptance of the assessment of the environment, emotions, autoimmune, functioning of the body, body image.

The quick access to the sentiment analysis on the image of one’s body, the emotions experienced and specific areas of difficulties for people in the risk group prone to anorexia, enables the specialists to diagnose and start treatment in a very short time. As a consequence, the prognosis for recovery can be better. According to our knowledge, no existing methods of processing the natural language for automatic language analysis to support psychologist’s work are known. Most of the research found in the literature focuses mainly on the patient’s statements. The proposed research, on the other hand, intends to determine the quantitative analysis of the patient’s health condition based on his/her notes (statements), which is a complete novelty.

Qualitative and statistical evaluation of sentiment analysis results showed statistically the significant compliance of the results for both proposed methods: the dictionary method and RNN with the opinions of the expert. The RNN method obtained better, not statistically significant, the value of the measure F1-score, with different amounts of measures: TPR, TNR, PPV, NPV. In the future, some results from presented studies can be used to build a complex classifier that uses dictionary and RNN methods as component predictors.

The results of analysis referring to emotions indicate that it is impossible to determine the level of intensity of a given emotion for an individual patient. It is because sentences are short and do not contain words occurring in the dictionaries. Therefore, it is required to collect more statements for a particular patient in the future.

Automatic detection of 6 specific areas of difficulty indicated by psychologists seems to be a valuable initial hint for the therapist. Here, the effectiveness depends on the number of terms stored in dictionaries. The authors, due to the pilot nature of the study, focused on assigning terms from the existing sentiment dictionary to specific difficulty areas, created by Wilson, Wiebe, and Hofman. In the future, the list of terms assigned to specific areas of difficulty can be expanded, mainly by increasing the database of patients’ notes and referring to their medical history.

## Methods

**Fig. 4:**
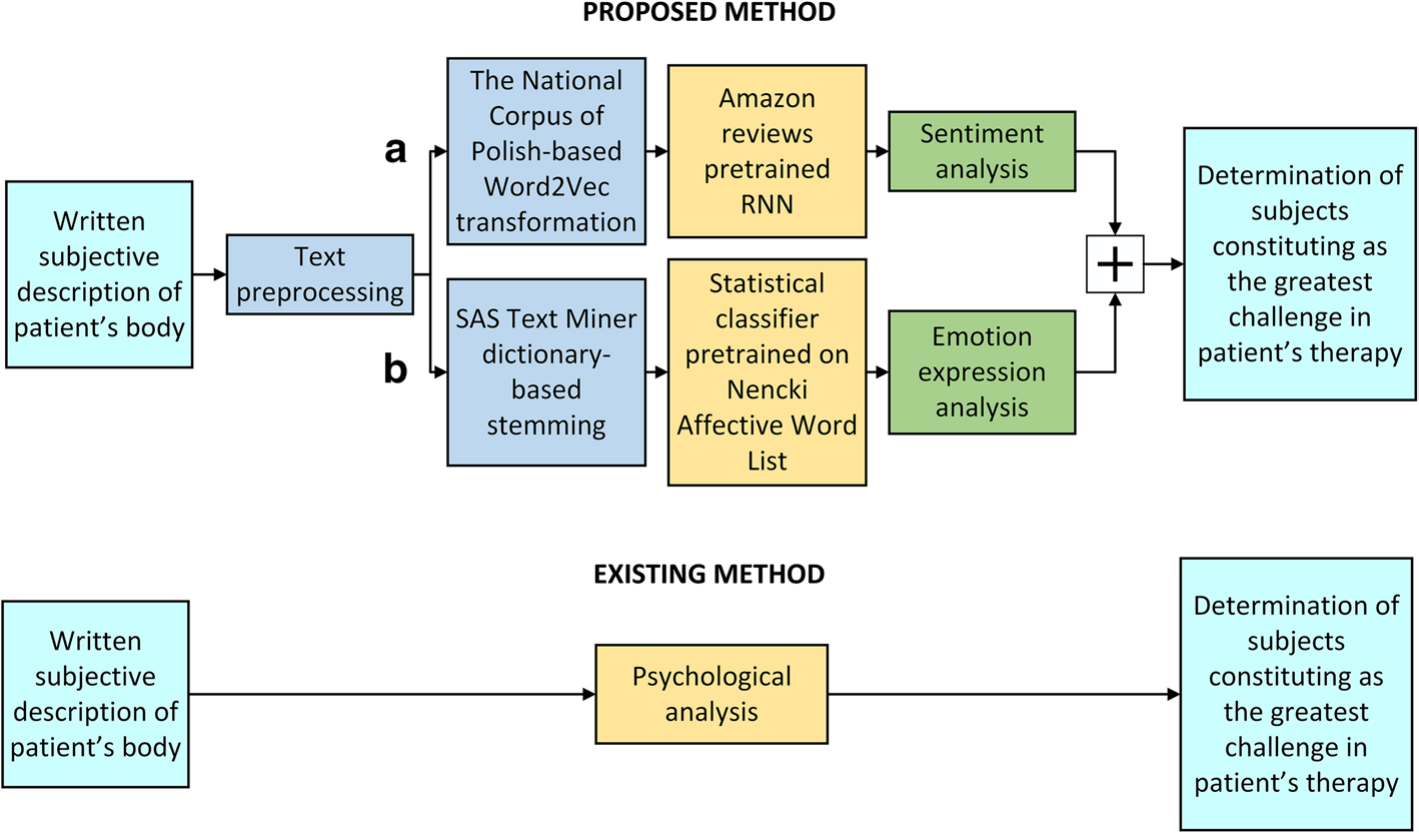
The diagram of the proposed method of computer-aided therapeutic diagnosis for anorexia: **a** sentiment analysis based on RNN, **b** statistical emotion classifier using Nencki Affective Word List

The goal of the presented method is to support a therapist in verifying the detailed hypotheses, proposed for a particular patient. Based on the literature review and our previous research, the method was proposed (Fig. 4). It consists of the following steps:

1. Preparing the free statement by a patient on his/her body image.
2. The sentiment analysis of the prepared statement.
3. The analysis of the patient’s emotions.
4. The analysis of particular areas of difficulties (from the previous authors’ paper [22]).

The first component consists of Recurrent Neural Network (RNN), pre-trained on Stanford Amazon reviews dataset [23], that allows for sentiment analysis of the patient’s body self-description (Fig. 4a). The outcome gives information about the polarity of delivered description, which can be positive, neutral, or negative. The second component is a statistical classifier [24] that performs emotion expression analysis of body description (Fig. 4b). As a result, it gives the information about the amount of happiness, anger, sadness, fear, and disgust expression exposed in body description delivered by the patient. The presented approach provides a kind of support for the existing method performed as psychological analysis, which, unfortunately, is time-consuming and prone to human error.

Further part of the paper presents the description of the proposed method for computer-aided therapeutic diagnosis of anorexia. First, the patients are asked by psychologists to write a subjective description of their body. Next, the written portrayal is converted, using Natural Language Processing, into a form of data that is interpreted by the computer for text analysis and machine learning tasks. Punctuation marks are removed. In the sentiment analysis, pathway words of the text data are then transformed into 100-dimensional numerical vectors based on The National Corpus of Polish using Word2Vec transformation. Then, the vectors are provided into the input of the Recurrent Neural Network, and description polarity is presented. The presentation of the emotion analysis pathway is as follows. Words are stemmed using the SAS Visual Text Analytics dictionary leading to separation of most frequent terms presented in body description. This step is important because some words have the same meaning but different lexical forms. These terms are provided into the statistical classifier, and as a result, the amount of certain emotions in the patient’s body description is shown.

### The free statement of patients’ body image

Having considered all the obstacles the anorexia patients encounter during the medical review, the psychologist asks the patient to write a short statement about his/her body. The following criteria for including patients into the research group were adopted:

- The age of the patients corresponds to the adolescence (12–18 years old),
- The initial diagnosis of anorexia made by psychiatrist,
- The lack of other coexisting psychical disorders,
- The disorder lasts no longer than 3 years.

The following criteria for excluding the patients from the research group are:

- The patients cover other age groups,
- The lack of circumstances for an initial diagnosis of anorexia made by psychiatrist,
- The presence of other coexisting physical disorders, for instance, depression,
- The duration of the disorder is longer than 3 years.

The following criteria for including patients into the control group were adopted:

- The age of the patients corresponds to the adolescence (12–18 years old),
- The lack of psychical disorders.

The research material contains the collection of free statements on “The image of my body”:

- The collection of 44 statements of people with the diagnosed anorexia,
- The collection of 52 statements of healthy people.

### The research and control group

Within the developed criteria, 44 girls with anorexia (restrictive form) were included in the research group. The participants were aged 12–18, an average of 15.1 ± 2. The anorexia was diagnosed according to ICD-10 and DSM IV criteria. The average weight of the girls was 37.2 ± 6.1 kg, and BMI was between 11.1 and 20.6, average 15.6 ± 2.4 (*p* < 0.001 vs. control group), and BMI SDS from − 5.2 to 0.9, average − 2.63 ± 1.28 (*p* < 0.001 vs. control group).

The control group consisted of 52 healthy girls, aged 12–18, average 14.9 ± 1.6, the average weight was 55.3 ± 9.8 kg and BMI from 16.1 to 24.8, average 20.1 ± 2.1, and BMI SDS from − 2.6 to 2.6, average 0.09 ± 1.16. The participants in the control group were female students of primary, middle, and secondary schools in the city of Gliwice.

The girls in the research group, with the diagnosed anorexia, had statistically lower BMI values comparing to those in the control group (*p* < 0001). The BMI values indicate the severe underweight of girls in the research group and the normal weight of girls in the control group. The BMI value depends on sex and changes, along with a person’s age. Therefore, for assessing the nutritional status of the girls, the normalized values were also presented using the Standard Deviation Score (SDS). According to the normalized standards for age and sex, the value below − 1 stands for underweight, values from − 1 to 1 stand for average weight, and 1–2 indicate overweight, the value more than 2 of SDS indicates severe obesity [25, 26]. BMI values for girls in the research group were statistically lower than the values of healthy girls–control group (*p* < 0.001), which proves the severe underweight of girls suffering from anorexia and average weight of the girls in the control group.

### Sentiment analysis of the statement

The methodological approach considers the sentiment analysis as supervised or no supervised classification depending on what kind of documents, re-classified as positive and negative, are accessible. In the unsupervised approach, the dictionary methods are applied [27–30]. In the supervised approach, the algorithms of machine learning, such as artificial neural networks [31–34], Support Vector Machine [35] are used in order to find the dependencies between the features of the text excerpt and the opinion expressed in a document. An expert in a particular area must prepare the required training set.

The supervised classification is usually conducted at the level of the entire document. Lots of research indicates the high effectiveness of Bayes classifiers [36–38] and the Support Vector Machine [39, 40]. However, the appropriate choice of classifier input variables is still the issue to be solved. Commonly, the desired input variables are the selected terms, their weight, and normalized frequencies, tags describing part of the speech, opinion-forming words, and the occurrence of negative words.

If the sentiment analysis encounters the problems with the classification of a sequence of words with various lengths, then the RNN can be used. This approach focuses on avoiding exploding and vanishing gradients. In the first case, it can lead to the fluctuation of neural weights values during the learning phase; in the second case, to the overextended time of training with the minimum effectiveness achieved. The Long Short-Term Memory cells (LSTM), which is the most commonly used solution, is applying to avoid such problem [41].

**Fig. 5:**
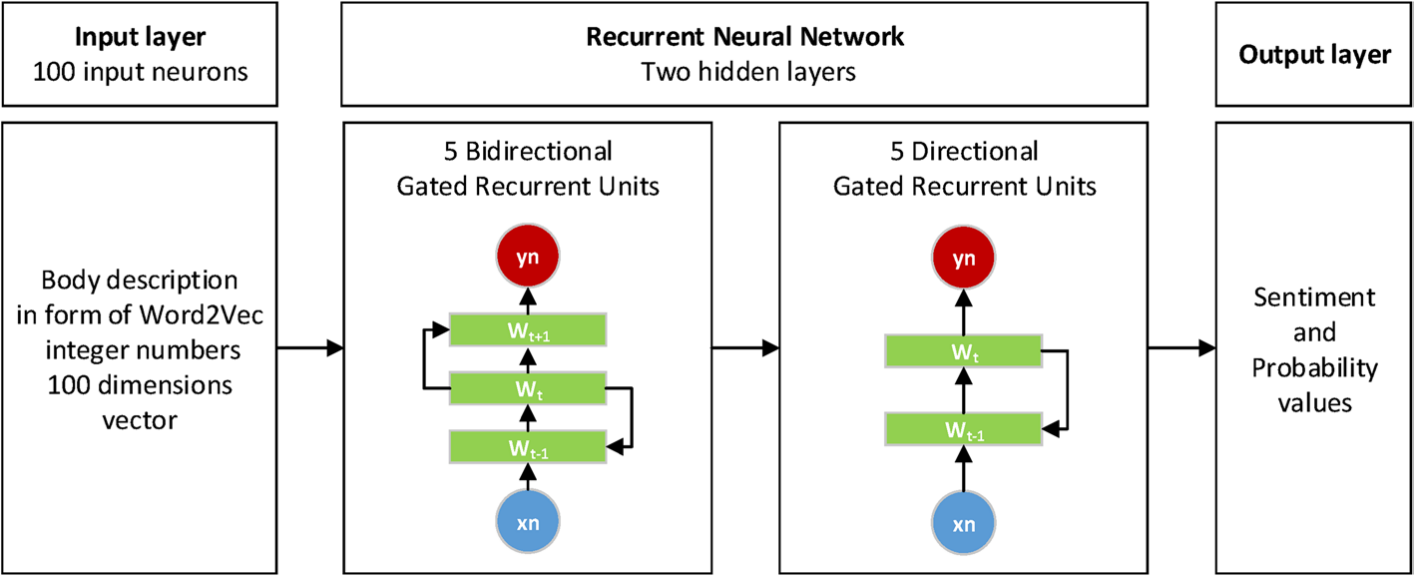
The outline of the applied RNN network for sentiment analysis of the entire document

In the proposed attitude, we decided to develop a method of sentiment analysis for the entire document using the RNN with 2 layers of Gated Recurrent Units [42]. The outline of the applied network is presented in Fig. 5. The architecture is taken from the SAS Visual Text Analytics example—Sentiment Analysis using DeepRNN [43].

The possibility of using knowledge transfer in machine learning explains why we decided to choose the presented approach. The collection of free statements on the body image was small compared to the number of parameters of the model based on Artificial Neural Network. Hence the research applied a trained model on the collection of opinions concerning the Stanford Amazon Dataset service [13] and then trained the selected model on a specific text corpus.

The Stanford Amazon review dataset contains 34,686,770 reviews that have been used for pre-training of the RNN. Reviews include product and user information, ratings, and a plaintext review. The polarity of the review was based on users’ scoring value. Reviews provided into the presented neural network were translated into Polish and transformed with Word2Vec approach so that they would correspond to the inputs of the RNN.

The Deep Learning method provided in the presented approach uses the RNN. The input layer of the network contains 100 neurons corresponding to the 100-dimension vector representation of words.

The presented network contains two hidden layers. The first layer is made up of 5 Bidirectional Gated Recurrent Units, which contain two subunits, one for forward states and another for backward states. It gives excellent results and provides a better understanding of the context. The second hidden layer contains 5 directional Gated Recurrent Units [42], which are only able to store representations of recent input events in the form of activations. The output layer provides information about sentiment values and the probability of achieved sentiment values.

**Fig. 6:**
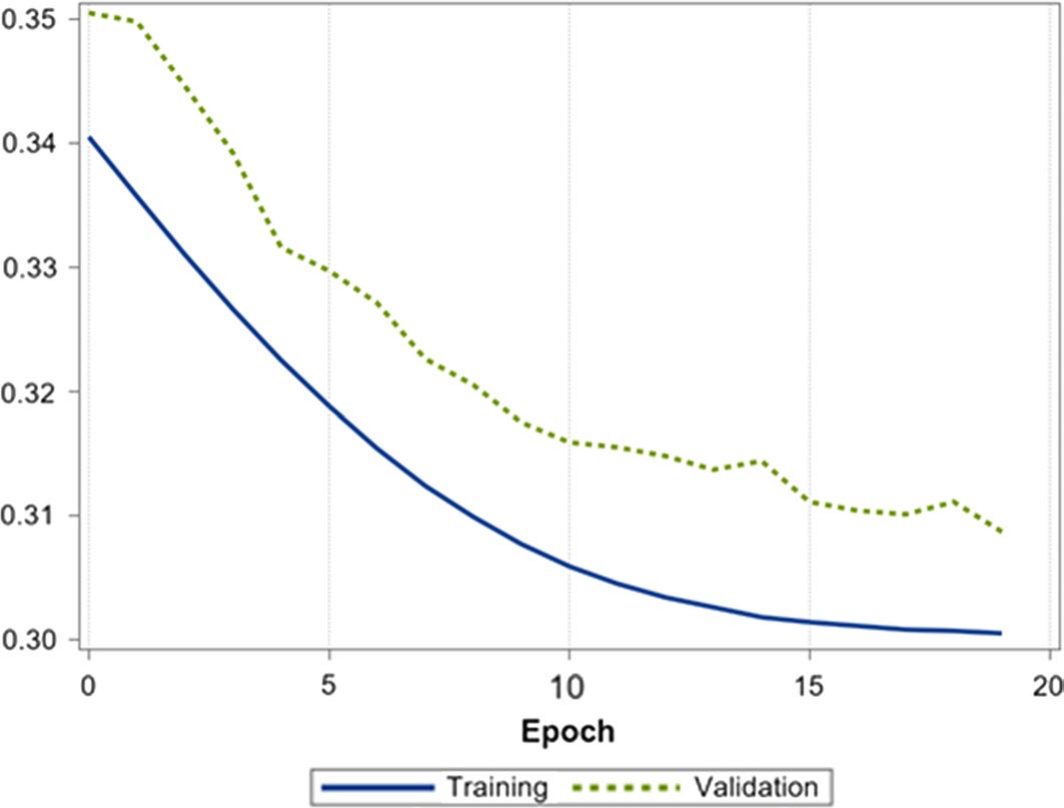
Learning curves for training and validation data set

The optimization tasks are performed using Adam optimizer, also called the Adaptive Moment Estimation. This method allows for efficient stochastic optimization and only requires first-order gradients with little memory requirement [44]. The optimizer computes individual adaptive learning rates for different parameters from estimates of first and second moments of the gradients. Optimal parameters of the Adam optimizer were beta1 = 0.9, beta2 = 0.999 and learning rates 0.000005. We are using Categorical Cross Entropy (CCE) as common loss function in neural network classification tasks, which punishes all misclassifications equally. CCE assumes that only one class is correct and is defined as:

1 $$ CCE = - \frac{1}{N}\mathop \sum \limits_{i = 1}^{N} { \log }\left( {p_{i} \left[ {y_{i} } \right]} \right), $$

where *N* is the size of the training dataset and $p_{i} \left[ {y_{i} } \right]$ is the probability vector output of the network at the target index $y_{i}$ for the $i{\text{th}}$ example. Figure 6 presents the learning curves for training and validation data set.

### Assessment of patient’s emotions

**Fig. 7:**
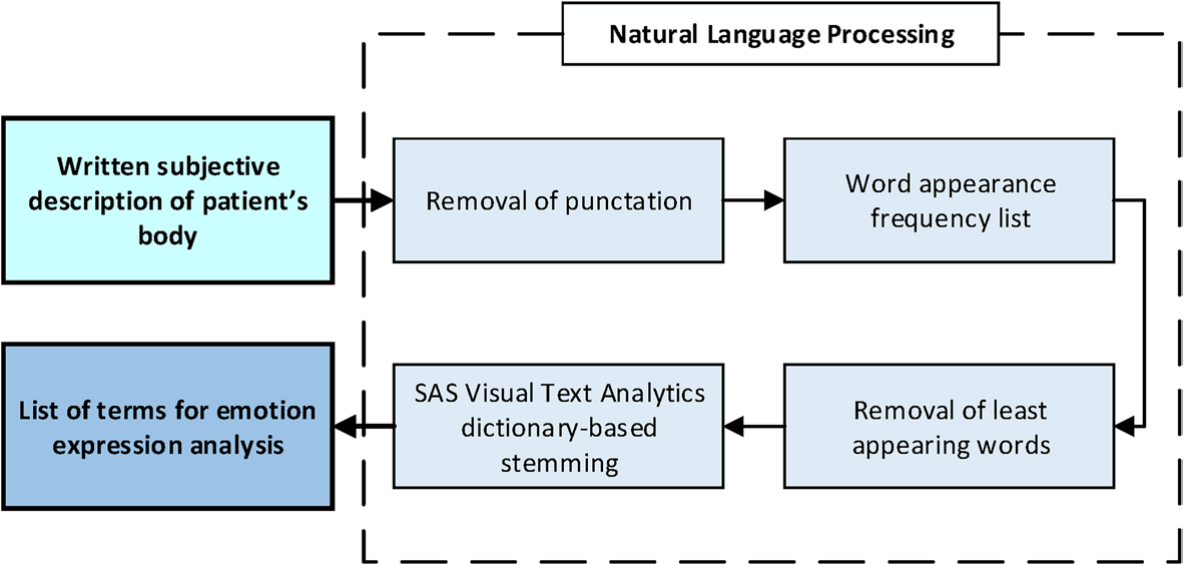
The schema of document processing phases into the list of words into their basic grammatical form

The presented approach presents the techniques of the initial processing taken from Natural Language Processing. In further analysis, the document is presented as a vector, where particular elements inform which words occur the most frequently. Forming the initial corpus of the document to the desired form requires (Fig. 7):

- removing punctuation marks at beginning and compiling the occurrence a list of words, individually for each document,
- removing words negligible for further analysis (those words form so-called stop-list),
- transferring the words occurring in the list into their basic grammatical form (*stemming*).

Depending on the language specificity, word transformation into their basic form can be achieved thanks to the rules or dictionaries. The words, which remained after the initial document transformation, are labeled as *terms.* Here, the dictionary approach focused on the Polish language dictionary included in SAS Visual Text Analytics was used [45].

In the next phase of the analysis, Nencki Affective Word List (NAWL) [46] was used. It consists of 2902 Polish words and their ratings connected to different aspects of expressing emotions. The database is a Polish adaptation of the Berlin Affective Word List-Reloaded (BAWL-R), commonly used to investigate the affective properties of German words. Affective, normative ratings were collected from 266 Polish participants (136 women and 130 men) [46].

**Fig. 8:**
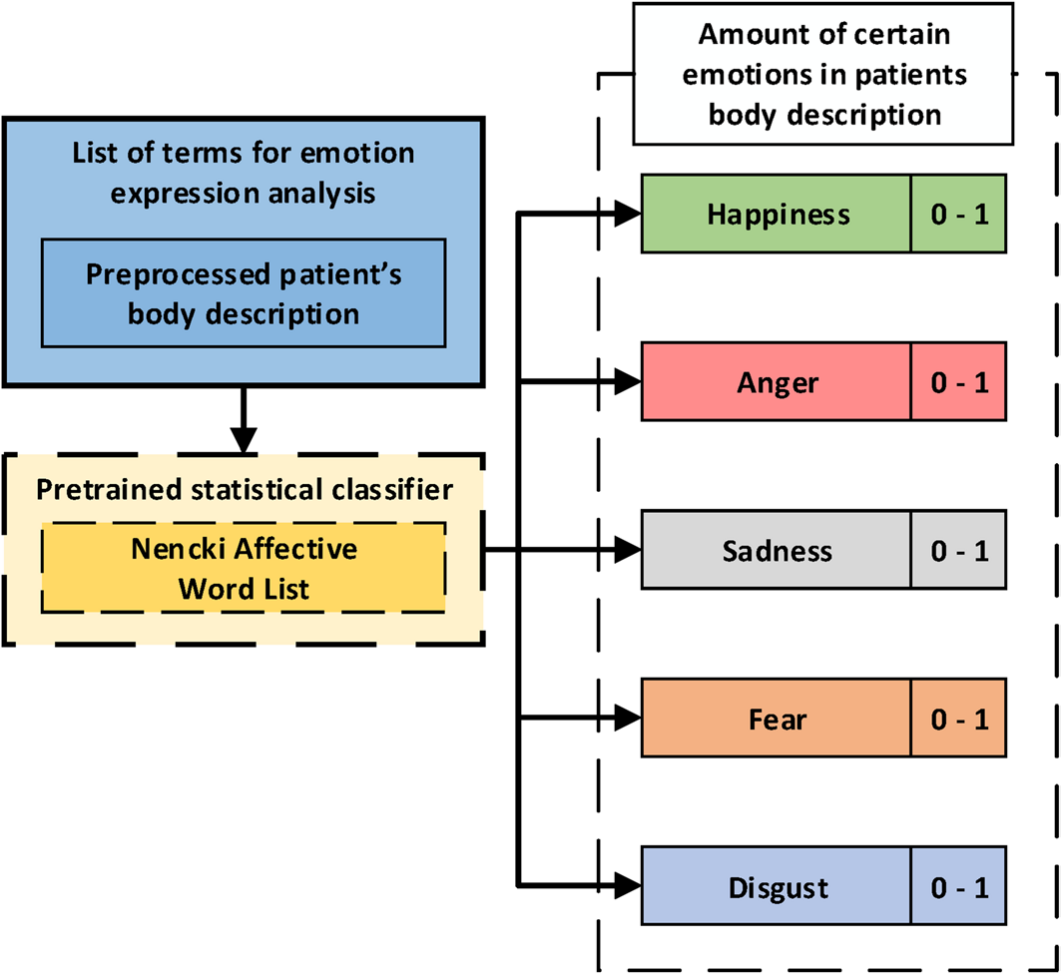
The schema of taxonomy used for determining the intensity of five basic emotions

We developed one-level taxonomy to ensure the analysis of emotional intensity in particular statements supported by the mentioned dictionary (Fig. 8). It includes 5 groups, and each refers to the particular basic emotion: happiness, anger, sadness, fear, and disgust. The rules that determine the affiliation to the particular group of emotions are the measures of emotional sentiment taken from the NAWL dictionary. The developed taxonomy uses a dictionary approach and the statistical model presented and discussed by Ningham et al. [47].

### The assessment of particular areas of difficulties

The previous research showed the relationship between the vocabulary used in the patient’s statement and the morbidity. The proposed method intends to provide additional support for the psychologist by developing 6 detailed categories corresponding to the patient’s areas of difficulties [22]:

- Self-esteem: an esthetic way of perceiving the body,
- Acceptance of the social assessment: the reception of the person perceiving it by society,
- Emotions: experienced emotions,
- Autoimmune: descriptions of aggressive and self-aggressive behaviors,
- The functioning of the body: description of the functioning of the body,
- Body image: the image of individual parts of the body.

The presented approach used document presentation in the form of words list, where the words occur in their basic form. The initial words processing into their basic grammatical form were achieved in the stage, referring to the patient’s emotion assessment.

The development of the detailed dictionaries was based on the sentiment, created by Wilson, Wiebe, and Hofman [48], however, the particular categories were created by psychologists—experts specializing in anorexia diagnostic and therapy [22].

### The conducted experiments

The following experiments were conducted during the research:

#### Experiment 1 (E1)

1. The sentiment label for statements included in the corpus, (presented in section “The free statement of patients’ body image”), was developed together with the psychologists.
2. The selected RNN architecture was trained on the collections depicted in section “Sentiment analysis of the statement.”
3. The scoring of the sentiment analysis of a statement by comparing the results from RNN and labels developed by experts.
4. The obtained results were compared also to sentiment analysis ones, based on the dictionary method presented in [11].

#### Experiment 2 (E2)

1. The statements from the corpus (see section “The free statement of patients’ body image”) were processed into their basic grammatical form according to the method depicted in section “Assessment of patient’s emotions”.
2. The analysis of processed notions for occurring five basic emotions: happiness, anger, sadness, fear, and disgust was made.

#### Experiment three (E3)

1. The statements from the corpus (see section “The free statement of patients’ body image”) were processed into their basic forms according to the method depicted in section “Assessment of patient’s emotions”.
2. The dictionary analysis was performed for occurring particular areas of difficulties according to six detailed developed dictionaries (see “The assessment of particular areas of difficulties” section).

### The quality evaluation

To evaluate the classification quality, the following measures were introduced:

True-positive rate (TPR): ${\text{TPR}}\;{\text{ = }}\;\frac{{{\text{TP}}}}{{{\text{TP}}\;{\text{ + }}\;{\text{FN}}}}$,

True-negative rate (TNR): ${\text{TNR}}\;{\text{ = }}\;\frac{{{\text{TN}}}}{{{\text{TN}}\;{\text{ + }}\;{\text{FP}}}}$,

Positive predictive value (PPV): ${\text{PPV}}\;{\text{ = }}\;\frac{{{\text{TP}}}}{{{\text{TP}}\;{\text{ + }}\;{\text{FP}}}}$,

Negative predictive value (NPV): ${\text{NPV}}\;{\text{ = }}\;\frac{{{\text{TN}}}}{{{\text{TN}}\;{\text{ + }}\;{\text{FN}}}}$,

F1 measure: $$  {\text{F1}}\;{\text{ = }}\;{\text{2}}\frac{{{\text{PPV}}\;{\text{*}}\;{\text{TPR}}}}{{{\text{PPV}}\;{\text{ + }}\;{\text{TPR}}}}\;{\text{ = }}\;\frac{{{\text{2TP}}}}{{{\text{2TP}}\;{\text{ + }}\;{\text{FP}}\;{\text{ + }}\;{\text{FN}}}}{\text{,}} $$

where

TP—true positive, the number of true-positive predictions,

TN—true negative, the number of true-negative predictions,

FP—false positive, the number of false-positive predictions,

FN—false negative, the number of false-negative predictions.

The results of the sentiment analysis were subjected to statistical evaluation. Statistical analysis of results provided the information whether the median value of expert assessment coincides with the median of the results obtained by the dictionary and RNN methods and whether the medians of both methods overlap (H0 hypothesis: equality of medians). For this purpose, the Wilcoxon matched-pairs signed-ranks test was used.

## Data Availability

The data that support the findings of this study are available from corresponding author.

## Abbreviations

AN: Anorexia nervosa
BAWL-R: Berlin Affective Word List-Reloaded
FN: False negative
FP: False positive
GRU: Gated Recurrent Unit
LSTM: Long short-term memory
NPV: Negative predictive value
PPV: Positive predictive value
RNN: Recurrent Neural Network
TNR: True-negative rate
TN: True negative
TP: True positive
TPR: True-positive rate
